# Antibiotic-Resistant Pathogenic *Escherichia Coli* Isolated from Rooftop Rainwater-Harvesting Tanks in the Eastern Cape, South Africa

**DOI:** 10.3390/ijerph15050892

**Published:** 2018-05-01

**Authors:** Mokaba Shirley Malema, Akebe Luther King Abia, Roman Tandlich, Bonga Zuma, Jean-Marc Mwenge Kahinda, Eunice Ubomba-Jaswa

**Affiliations:** 1 Council for Scientific and Industrial Research, Natural Resources and the Environment, P.O. Box 395, Pretoria 0001, South Africa; jeanmarcmk@yahoo.co.uk; 2 Antimicrobial Research Unit, College of Health Sciences, University of KwaZulu-Natal, Private Bag X54001, Durban 4000, South Africa; lutherkinga@yahoo.fr; 3 Faculty of Pharmacy, Pharmaceutical Chemistry Division, Rhodes University, Grahamstown 6140, South Africa; roman.tandlich@gmail.com (R.T.); b.zuma@ru.ac.za (B.Z.); 4 Department of Biotechnology, University of Johannesburg, 37 Nind Street, Doornfontein 2094, South Africa; euniceubombajaswa@yahoo.com; 5 Water Research Commission, Lynnwood Bridge Office Park, Bloukrans Building, 4 Daventry Street, Lynnwood Manor, Pretoria 0081, South Africa

**Keywords:** antimicrobial resistance, pathogenic *E. coli*, harvested rainwater, public health, Sub-Saharan Africa, alternative water source

## Abstract

Although many developing countries use harvested rainwater (HRW) for drinking and other household purposes, its quality is seldom monitored. Continuous assessment of the microbial quality of HRW would ensure the safety of users of such water. The current study investigated the prevalence of pathogenic *Escherichia coli* strains and their antimicrobial resistance patterns in HRW tanks in the Eastern Cape, South Africa. Rainwater samples were collected weekly between June and September 2016 from 11 tanks in various areas of the province. Enumeration of *E. coli* was performed using the Colilert^®^18/Quanti-Tray^®^ 2000 method. *E. coli* isolates were obtained and screened for their virulence potentials using polymerase chain reaction (PCR), and subsequently tested for antibiotic resistance using the disc-diffusion method against 11 antibiotics. The pathotype most detected was the neonatal meningitis *E. coli* (NMEC) (*ibeA* 28%) while pathotype enteroaggregative *E. coli* (EAEC) was not detected. The highest resistance of the *E. coli* isolates was observed against Cephalothin (76%). All tested pathotypes were susceptible to Gentamicin, and 52% demonstrated multiple-antibiotic resistance (MAR). The results of the current study are of public health concern since the use of untreated harvested rainwater for potable purposes may pose a risk of transmission of pathogenic and antimicrobial-resistant *E. coli.*

## 1. Introduction

Several countries around the world, including South Africa, make use of harvested rainwater (HRW) to meet their daily water needs. However, the most significant issue relating to the use of harvested rainwater is the potential health risk associated with the presence of various pathogenic organisms in such water [1]. Indicator organisms like *E. coli* have been used to determine the microbiological safety of water meant for drinking and other human needs. Although most *E. coli* strains are non-pathogenic, certain strains may be pathogenic and carry virulence genes (VGs) [2]. Pathogenic *E. coli* strains which can cause diseases in both humans and animals are categorised as intestinal pathogenic *E. coli* (InPEC) and extraintestinal pathogenic *E. coli* (ExPEC) [3]. Intestinal strains are mostly referred to as diarrhoeagenic *Escherichia coli* (DEC) due to their ability to cause diarrhoea using diverse mechanisms [4]. The ExPEC strains have been reported to cause diseases such as urinary tract infections, neonatal meningitis, sepsis and wound infections and some examples include neonatal meningitis *Escherichia coli* (NMEC) and uropathogenic *E. coli* (UPEC) [3].

Six groups of DEC strains known to cause intestinal infections include enterotoxigenic *E. coli* (ETEC), enteropathogenic *E. coli* (EPEC), enterohemorrhagic *E. coli* (EHEC), enteroaggregative *E. coli* (EAEC), diffusely adherent *E. coli* (DAEC) and enteroinvasive *E. coli* (EIEC). Among all *E. coli* pathotypes, ETEC strains cause a cholera-like diarrhoeal disease and are the most common cause of childhood and travellers’ diarrhoea in developing countries [5]. Diffusely adherent *E. coli* pathotypes were previously implicated in intestinal infections (diarrhoea in children between the ages of 18 months and 5 years) and extraintestinal infections (urinary tract infections and pregnancy complications) [6]. EIEC shows pathogenic phenotypic and genetic similarities with *Shigella* spp. and can be identified by their epithelial cell invasiveness mediated in part by the *ipaH* and *virF* genes and association with dysentery [7]. EHEC is associated with bloody diarrhoea and haemolytic uremic syndrome and expresses one or two Shiga-like toxin-encoding genes *stx1* and *stx2* [8].

Several virulence genes in these *E. coli* pathotypes are responsible for a wide array of infections such as diarrhoea or haemolytic colitis, neonatal meningitis, nosocomial septicaemia, haemolytic-uraemic syndrome and urinary tract infections [9]. Current molecular-based techniques such as polymerase chain reaction (PCR) allow for the identification of these VGs by amplifying specific target regions [10]. Virulence genes associated with these pathogenic strains have been isolated in diverse environments in South Africa. For example, the presence of DEC virulence genes in 60% of samples collected from the Apies River (water and sediments) was reported by Abia et al. [11]. In another study, a high prevalence of virulence genes associated with four pathogenic *E. coli* types (EAEC, EHEC, EPEC, and EIEC) in domestic rainwater harvesting tanks in Kleinmond, Cape Town was documented by Dobrowsky et al. [12]. Apart from being pathogenic, some of these microorganisms have developed resistance to many of the drugs designed to treat the infections they cause. For example, the antimicrobial resistance patterns of *E. coli* isolates in outpatient urinary tract infections in South Africa was studied and the results revealed that the isolated *E. coli* were resistant to trimethoprim-sulfamethoxazole (TMP-SMX; 68%), amoxicillin (65%) and ciprofloxacin (41%) [13]. Another study focused on the hospital, and community isolates of uropathogens at a tertiary hospital in South Africa and results revealed that the most isolated bacterial pathogen was *E. coli* (39%) [14]. Furthermore, levels of *E. coli* resistance to amoxicillin and co-trimoxazole ranged from 43–100% and 29–90%, respectively. The presence of such drug-resistant bacteria in human settings has placed constraints on the choice of safe, effective and inexpensive antibiotics, especially for low- and middle-income countries [15]. As such, the progression of resistant bacteria and the increasing incidence of antibiotic resistance genes (ARGs) are thus of significant public health concern [16].

Although studies have been carried out on the presence of virulence genes and antibiotic-resistant bacteria in various water sources such as wastewater effluents, taps, wells and boreholes in South Africa, very few studies have investigated their presence in harvested rainwater [12,17,18,19]. This study aimed at reporting on the prevalence of pathogenic *E. coli* strains and their antibiotic resistance patterns in harvested rainwater collected from tanks in the Eastern Cape Province of South Africa. Such results would highlight the need for appropriate development and implementation of effective household water treatment methods, thereby protecting the lives of populations using such water for their daily needs. Moreover, results of the current study will also add to existing research databases which report on the circulating strains of antimicrobial-resistant organisms.

## 2. Materials and Methods

### 2.1. Study Site and Sample Collection

Rooftop-harvested rainwater samples were collected from 11 rainwater-harvesting systems situated at various sites around Grahamstown west, Rhodes University campus and Kenton-on-sea in the Eastern Cape Province, South Africa. The distance between Rhodes University (33°31’36” S, 26°51’63” E) and Grahamstown west (33°18’36” S; 26°31”36” E) is approximately 4 km while the distance between Rhodes University and Kenton-on-sea (33°42’0” S, 26°41’0” E) is about 59.2 km. Mean annual rainfall in Grahamstown is 650 mm, with bimodal peaks in October–November and again in March–April. All the sites were selected based on the diversity in environmental conditions (e.g., presence of foliage and birds) as well as the various uses of the water stored in the tanks. A total of 110 water samples were collected from the 11 selected tanks from June 2016 to September 2016 and tested for *E. coli.* Sterile 5 L bottles were used to collect rainwater samples weekly by first rinsing the tap connected to the tanks with 70% ethanol and letting the tap run for 30 s before collection. Rainwater samples were taken from the same tanks once a week. Samples were then transported to Rhodes University laboratory on ice for microbial analysis within 6 h.

### 2.2. Enumeration and Isolation of *E. coli*

Enumeration of *E. coli* was carried out using the Colilert-18^®^ Quanti-tray^®^/2000 (IDEXX Laboratories, Inc., Johannesburg, South Africa). The test was performed following the manufacturer’s instructions. After incubation at 37 °C for 18–24 h, presumptive *E. coli* isolates were obtained from fluorescent quanti-tray wells as described by Abia et al. [20]. The Colilert method has a detection limit ranging from <1 MPN/100 mL to >2419.6 MPN/100 mL. *E. coli* ATCC^®^ 25922 was used as a positive control and *Pseudomonas aeruginosa* ATCC 49189 as a negative control. One hundred (100) *E. coli* isolates were then selected from the various tanks. Of the 100 isolates selected, 66 isolates were chosen from T1-T6 (11 isolates from each tank), 20 isolates were from T7 and T8 (10 isolates from each tank) and 14 isolates from T9 and T11. T10 was excluded from further analysis due to poor growth of the selected isolates from the culture media.

### 2.3. Identification of Pathogenic *Escherichia coli* Strains Using Polymerase Chain Reaction (PCR)

#### DNA Extraction and Detection of Virulence Genes in *E. coli* Isolates

One hundred (100) presumptive *E. coli* isolates were randomly selected and inoculated separately into 5 mL Erlenmeyer flasks containing 2 mL nutrient broth (Merck, Johannesburg, South Africa). The flasks were incubated overnight at 37 °C on a rotary shaker at 100 rpm. DNA was extracted from 1 mL of the overnight culture using the InstaGene^TM^ Matrix (Bio-Rad Laboratories, Johannesburg, South Africa) following the manufacturer’s instruction. The template DNA was stored at −20 °C for PCR assays. All selected samples were first confirmed as *E. coli* by testing for the presence of the malate dehydrogenase (*mdh*) gene which is found in most *E. coli* strains [21]. After that, the presence of a total of eight VGs (*eaeA* (EPEC/EHEC), *eagg* (EAEC), *ipaH* (EIEC), *ST* (ETEC), *ibeA* (NMEC), *stx1* (EHEC), *stx2* (EHEC) and *flicH7* (EHEC)) were investigated. The primer sequences and the PCR-cycling conditions for the identification of the various VGs were as previously described by Abia et al. [19]. Both multiplex and singleplex PCR assays were performed for the target genes. Multiplex PCR assays were divided into 3 sets where set 1 contained *eaeA*, *eagg* and *ipaH*, set 2 contained *flicH7* and *Stx1* and finally set 3 contained *ST* and *ibeA* genes [19,22,23]. Singleplex real-time PCR assays were performed for the *mdh* and *stx2* target genes [24,25].

### 2.4. Screening for Antibiotic-Resistant *E. coli*

The remaining 1 mL from the overnight culture was used for antibiotic resistance analysis using the disk-diffusion method [26]. Briefly, 100 µL of overnight *E. coli* culture was spread on Mueller–Hinton agar (Lasec, Cape Town, South Africa) and antibiotic mastrings (Davies diagnostics, Johannesburg, South Africa) were carefully placed onto inoculated plates, incubated at 37 °C for 18–20 h. Following incubation, the diameters (in millimetres) of clear zones of growth inhibition around the antibiotic disks were measured using a ruler and compared with the Clinical Laboratory Standard Institute (CLSI) 2013 reference values. The different phenotypic profiles (resistant, intermediate or susceptible) of the isolates were then determined following the interpretation of the zones of inhibition. A total of 11 antibiotics were selected for this study (Table 1). The antibiotics were chosen for their frequent use in the treatment of bacterial infections in South Africa Both positive (*E. coli* strain ATTC 25922) and negative controls (*E. coli* strain ATTC 35218) were included in the experiments.

### 2.5. Data Analysis

Data were analysed using the Statistical Package for the Social Sciences (SPSS) (Version 16.0, Prentice Hall Press Company, NJ, USA) [27]. The *E. coli* counts were log_10_ transformed before computation of the means and standard deviations. A multiple antibiotic resistance (MAR) index was performed following the procedure described by Krumperman [28]. A MAR index for an isolate was calculated using the formula: MAR = a/b where ‘a’ is the number of antibiotics from each group to which a particular isolate was resistant and ‘b’ is the total number of antibiotics against which the isolate was tested. A resistance index greater than 0.2 shows that *E. coli* isolates are likely to be from a high-risk source.

## 3. Results

### 3.1. Concentration of *E. coli* in Harvested Rainwater (HRW)

The log transformed (log_10_) *E. coli* counts and the mean *E. coli* counts in most probable number per 100 mL (MPN/100 mL) from individual tanks are shown in Table 2. The abundance of *E. coli* in the rainwater-harvesting tanks differed according to the location of the HRW system. The highest concentrations of *E. coli* were detected in tanks situated at Rhodes University (T1–T6).

### 3.2. Identification of Virulence Genes among *E. coli* Isolates

**Figure 1:**
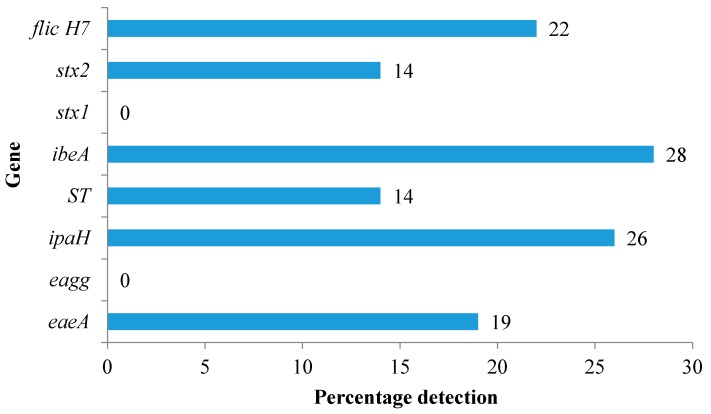
Overall prevalence of virulence genes in isolated *E. coli* from harvested rainwater (HRW) tanks.

Samples which generated fluorescence from the Quanti-tray^®^/2000 cells were selected for the identification of the *E. coli* VGs. The most detected pathotypes were the NMEC and EHEC while the least detected pathotype was EAEC (Table 3). Of the 100 isolates tested for the VGs, 28% were identified as *ibeA* positive (Figure 1). The EAEC pathotype (*eagg* gene) was not detected among the tested isolates. Similarly, the *Stx1* gene of EHEC was not detected in any of the isolates.

### 3.3. Antibiotic-Resistance Profiles of *E. coli* Isolated from the Harvested-Rainwater Samples

#### 3.3.1. Overall Antibiotic Resistance Profiles of the *E. coli*

**Figure 2:**
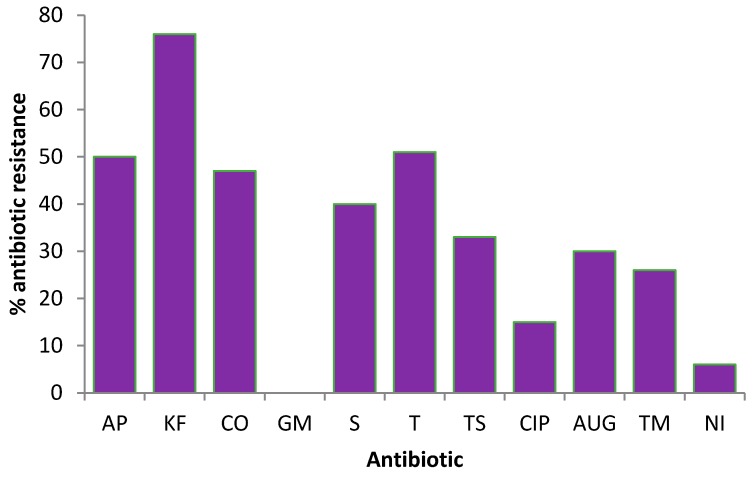
Percentage antibiotic resistance of *E. coli* isolates to selected antibiotics.

All the 100 *E. coli* isolates tested for the presence of VGs were further tested for antibiotic resistance. Of the 11 antibiotics tested, the highest resistance displayed by *E. coli* isolates was against Cephalothin (76%) while complete susceptibility (100%) was observed to Gentamycin. The overall percentage of antibiotic resistance found in the tested isolates is shown in Figure 2. *E. coli* isolates were resistant to 10 of the 11 antibiotics used in this study with the resistant rate ranging from 9% to 76%. Furthermore, a low percentage of the isolates showed resistance to Ciprofloxacin (15%) and Nitrofurantoin (9%).

The bacterial resistance rate in individual tanks is shown in Table 4. Resistance to Nitrofurantoin was only observed in T1 and T2, while resistance to Augmentin was seen in all the tanks studied. Some of the selected isolates showed the presence of multiple-antibiotic resistance (MAR) where simultaneous resistance ranged from 3 to 9 antibiotics.

#### 3.3.2. Prevalence of Multiple-Antibiotic Resistance

The presence of MAR was also observed for most isolates. Multiple-antibiotic resistance in this study was defined as the resistance of bacterial strains to three or more antibiotics [20]. Of the 100 isolates tested, more than half (52%) were MAR (Table 5). Ten of the 52 MAR isolates demonstrated simultaneous resistance to up to nine antibiotics. A total of 24 different MAR phenotypes were identified in this study.

## 4. Discussion

### 4.1. Concentration of *E. coli* in Harvested Rainwater

Faecal coliform bacteria such as *E. coli* have been widely used as indicator organisms to assess the possibility of pathogen presence in water [29]. Therefore, the presence of *E. coli* in roof-harvested rainwater in the Eastern Cape, South Africa, was monitored. All the 11 tanks monitored in this study were contaminated with varying concentrations of *E. coli* (0.85 ± 0.26–3.02 ± 0.21 MPN/100 mL)*.* Other scholars have previously reported on the high detection of *E. coli* from roof-harvested rainwater (2 to 986 CFU/100 mL; 1 to 99 MPN/100 mL and 0 to 41 CFU/100 mL) [1,30,31]. None of the tanks monitored in this study met the guidelines for drinking-water quality, as the *E. coli* amounts exceeded the South African drinking-water quality guidelines of 0 CFU/100 mL. The considerable amounts of *E. coli* in the harvested rainwater samples indicate possible faecal contamination.

The variations in the number of *E. coli* contamination in different HRW systems could be attributed to the fact that some of the HRW systems (Rhodes University) had a constant presence of birds which could have landed and dropped faecal matter on the roof, thereby contaminating tank water. Bird faecal droppings may negatively impact roof-harvested rainwater quality due to the presence of zoonotic pathogens [32]. A study conducted in South Africa investigated antibiotic resistance in *E. coli* isolates from roof-harvested rainwater tanks and urban pigeon faeces as the likely source of contamination and concluded that urban pigeons, the most likely source of HRW contamination, are also reservoirs of multiple antibiotic-resistant bacteria [33]. The findings of the South African study on bird faeces and antibiotic-resistant *E. coli* have a similar conclusion to our study where bird faecal matter was suspected to contribute to the contamination of HRW. In cases where the sources of faecal pollution in rainwater tanks are suspected to be from birds, the application of bird faecal markers may have the potential to confirm the sources of faecal contamination in a rainwater tank [32]. In another study to identify the likely sources of potential clinically significant *E. coli* in rainwater tanks, a source-tracking approach was used where a biochemical-fingerprinting method for typing of *E. coli* strains revealed that of the 43 strains from rainwater tank samples, 14 (from 7 tanks) and 9 (from 6 tanks) had identical biochemical phenotypes to those found in bird and possum faecal samples, respectively [34]. Furthermore, five strains from 4 rainwater tanks were identical to those isolated from both bird and possum faecal samples [34].

The rainwater tanks in the current study are used for various purposes such drinking and toilet flushing (for tanks situated at Rhodes University). Tanks situated at Grahamstown west were mainly used for gardening and sometimes drinking, depending on the availability of the municipal supply, while Kenton-on-sea tanks were used for indoor potable uses such dish-washing and laundry. In order to reduce or limit the risk of pathogenic and antimicrobial resistant *E. coli*, constant cleaning and maintenance of the catchment area may significantly improve the quality of the HRW, as the catchment area is suspected to contribute largely to the deterioration of the HRW in the Eastern Cape due to birds landing on the roof. Installation of first flush diverters may also help to improve the quality of the HRW. A study conducted in South Africa on the quality of HRW reported that 100% of the samples tested for *E. coli* exceeded the recommended standard of 0 CFU/100 mL [12]. Their results were similar to the ones observed in this study where all of the samples showed high levels of *E. coli*. In the Eastern Cape, where harvested rainwater is used for various household purposes including drinking, the presence of *E. coli* in the rainwater tanks is a major health concern as the presence of *E. coli* could imply the presence of other bacterial pathogens which may be detrimental to the health of rainwater users. The findings of the current study are of significant health concern as antibiotic-resistant pathogenic *E. coli* isolates may cause diseases if the users of the HRW consume the water without treatment. Furthermore, resistance of the isolated pathogenic *E. coli* to commonly used antibiotics in South Africa may lead to antibiotic treatment failure with serious public health implications for the population and the country.

### 4.2. Identification of Virulence Genes among *E. coli* Isolates

Pathogenic *E. coli* strains are a major cause of infections worldwide, the most common of which are diarrhoeal diseases. All the 100 *E. coli* isolates from the tanks tested positive for one or more VGs. The most detected pathotype was the NMEC (*ibeA*; 28%) which is responsible for neonatal meningitis and endothelial cell invasion [35]. The *ibeA* gene is also reportedly found in avian pathogenic *E. coli* (APEC) and causes avian colibacillosis, which is the most significant infectious bacterial disease of poultry worldwide [35]. The detection of the *ibeA*-positive strains in this study possibly indicates that the observed pathotype may be due to the presence of birds around the HRW systems. Although the present study did not investigate whether the *ibeA* gene detected was of human or avian origin, the presence of *ibeA*-positive isolates in the HRW systems is still of health concern given that there could be a possibility of zoonotic infections arising from the consumption of untreated rainwater containing these strains. Genes pertaining to other pathotypes of public health concern were also detected in the present study. For example, the *flicH7* (22%) and *Stx2* (14%) genes of EHEC were also detected in the isolates. Members of the EHEC group have been involved in many diarrhoeal disease outbreaks around the world, and they are known to cause hemorrhagic colitis and hemolytic uremic syndrome in humans [36].

The EHEC pathotype showed high prevalence across all the sampling sites except for the sites located in Grahamstown west. Both T1 and T6 which yielded a high percentage in VGs detection were situated at Rhodes University. Prevalence of the virulence gene *ipaH* (26%) (pathotype EIEC) was also noticeable in 4 tanks; 3 of the tanks were situated on campus and 1 in Kenton-on-sea. A previous study conducted in Cape Town, South Africa, reported that EPEC and EHEC (3% each) were detected in lower numbers, whereas EIEC was not identified in any of the rainwater tanks tested in their study [12]. The results differ from the findings of the current study where EIEC (26%) was the second most detected pathotype. This shows that the location of the tank could affect the pathotypes detected. Due to the detection of *E. coli* pathotypes in the current study, there is a great need to create awareness on household treatment technologies among users of HRW. Available treatment options which have proven to be successful in the treatment of HRW such as boiling, closed-couple solar pasteurizer, and solar disinfection (SODIS) can be used to decontaminate HRW [37,38,39]. In this study, all the rainwater tanks did not have any treatment option fitted, such as first-flush diverters and filters, except for T5 which had a chlorinator. However, due to limited maintenance of the rainwater-harvesting systems, the chlorinator in T5 was clogged in the middle of the sampling season and the *E. coli* counts increased going forward. The interruption of the treatment option observed in this study is also a clear indication of lack of proper maintenance of the HRW systems.

### 4.3. Detection of Antibiotic-Resistant *E. coli* in Harvested Rainwater

Results of the antibiotic-resistance profiling of the isolates from harvested rainwater analysed in the current study revealed that most of the *E. coli* isolates were resistant to the commonly prescribed antibiotics in South Africa. In areas such as the Eastern Cape where most of the population rely on harvested rainwater, exposure to antibiotic-resistant bacteria can further increase the health risk, particularly to children, the elderly and immune-compromised individuals. Antibiotic resistance is on the increase worldwide as most microorganisms now exhibit resistance to a large number of known antibiotics. The *E. coli* isolates from harvested rainwater in this study revealed resistance to Cephalothin (76%), Tetracyclines (51%), Colistin sulphate (47%), Ampicillin (50%) and Streptomycin (40%). The antibiotics most used in South Africa are the penicillins (Cephalothin) and fluoroquinolones, (Ciprofloxacin and glycopeptides) [40]. Tetracyclines and trimethoprim are also extensively used in the treatment of bacterial infections in both human and animals [41].

Cephalothin belongs to the β-Lactam class of antibiotics which are characterised by a β-lactam ring in their molecular structure [42]. Resistance to beta-lactam antibiotics has been highly documented as bacterial strains that produce extended-spectrum beta-lactamases have become more common [43]. Extended-spectrum beta-lactamase (ESBL)-producing *E. coli* are highly resistant to an array of antibiotics and infections by these strains are difficult to treat [43]. Furthermore, genes for ESBLs are most often encoded on plasmids, which can readily be transferred between bacteria [44]. Given that most of the isolates carrying virulence genes, especially the *ibeA* gene, were also resistant to Cephalothin, this could suggest that most of the isolated *E. coli* strains may carry the ESBL genes with the possibility of transfer to related organisms within the rainwater tanks. However, it is necessary to conduct further studies to ascertain such ARGs’ transfer within harvested-rainwater systems. Results of such studies would highlight the need for implementation of appropriate treatment options and better policies for the safe use of harvested rainwater, especially where such water is the main source of water for personal and household uses, thus protecting the lives of users of harvested rainwater.

In the current study, the tested *E. coli* isolates showed resistance to one or more antibiotics with the highest *E. coli* resistance recorded against Cephalothin, Ampicillin and Tetracyclines. Also, there was evidence of MAR *E. coli* in almost all the HRW systems with some isolates showing simultaneous resistance to a panel of up to nine antibiotics. These results indicate that in the case of infections occurring due to the consumption of contaminated harvested rainwater, treatment may fail because of the persistent resistance of the *E. coli* isolates detected in the HRW systems. A similar study carried out in Pretoria and Johannesburg, South Africa, showed that the resistances most encountered were against Ampicillin, Gentamicin, Amikacin and Tetracyclines [34]. These results were not in agreement with our findings, where *E. coli* isolates were resistant to Cephalothin and 100% susceptible to Gentamicin, although the same method and concentration was used for Gentamicin in both studies. The difference in antibiotic resistance results from the two studies could be attributed to the fact that roof-harvested rainwater samples were collected from different locations (Gauteng and Eastern Cape). Our findings were, however, similar to the those of Chidamba and Korsten [34] in that the authors also reported a substantial prevalence of MAR. All the isolates tested in this study showed a MAR index greater than 0.2, suggesting that a greater proportion of the isolates were likely to be from a high-risk source such as faecal material. These results and the differences observed with other studies could inform those implementing antibiotic-resistance surveillance schemes that would address different geographical locations. Also, the presence of MAR *E. coli* in harvested rainwater could pose a severe health risk to the public in general, as antibiotic resistance decreases the efficiency of antibiotics used in the treatment of infections. These findings are of major concern, as more households are now reported to be using harvested rainwater for their daily water needs.

## 5. Conclusions

Rainwater samples tested in this study showed contamination with varying concentrations of pathogenic *E. coli* strains. The outcome of the study further demonstrates that HRW tanks could serve as reservoirs for not only pathogenic but also antibiotic-resistant *E. coli* strains including MAR strains. These findings suggest that the tested harvested rainwater was not fit for human consumption and, therefore, should not be used for potable purposes without appropriate treatment. Furthermore, routine monitoring and treatment are essential to ensure that harvested rainwater is fit for intended use as well as to stimulate the need for strategies (e.g., maintenance of HRW systems, constant cleaning of the roof, and installation of first-flush diverters to minimise faecal contamination) that would prevent the spread of antibiotic-resistant bacteria.

## Figures and Tables

**Table 1:** Antibiotics used to determine antibiotic resistance of *E. coli* isolates.

Class	Antibiotic	Abbreviation	Concentration (µg)
β-Lactams	Ampicillin	AP	10
	Cephalothin	KF	5
Polypeptides	Colistin sulphate	CO	25
Aminoglycosides	Gentamicin	GM	10
Aminoglycosides	Streptomicin	S	10
Tetracyclines	Tetracycline	T	25
Folate pathway inhibitors	Cotrimoxazole	TS	25
Fluoroquinolones	Ciprofloxacin	CIP	5
Penicillin combination	Augmentin(amoxillin-clavulanate)	AUG	30
Sulfonamides	Trimethoprim	TM	5
Nitrofurans	Nitrofurantoin	NI	300

**Table 2:** Log transformed *E. coli* (MPN/100 mL) concentrations from various rainwater tanks.

Tank ID	*n*	Minimum	Maximum	Mean ± Standard Deviation
T1	11	2.55	3.29	3.02 ± 0.21
T2	11	1.95	3.11	2.62 ± 0.35
T3	11	2.58	3.29	2.84 ± 0.25
T4	11	1.64	2.89	2.52 ± 0.42
T5	11	0.79	3.00	2.18 ± 0.82
T6	11	2.53	3.04	2.88 ± 0.19
T7	10	1.73	2.96	2.36 ± 0.37
T8	10	1.78	2.41	2.09 ± 0.22
T9	7	0.61	3.04	1.71 ± 0.82
T10	10	0.3	3.19	1.57 ± 1.04
T11	7	0.61	1.12	0.85 ± 0.26

**Table 3:** Number of virulence genes detected from rainwater-harvesting tanks.

Tank Location	Tank ID	Number of *E. coli* Isolates Tested	*EaeA (EPEC/EHEC)*	*Eagg (EAEC)*	*ipaH (EIEC)*	*ST (ETEC)*	*ibeA (NMEC)*	*Stx1 (EHEC)*	*Stx2 (EHEC)*	*flichH7 (EHEC)*
Rhodes University	T1	11	6 (55%)	0	4 (36%)	0	4 (36%)	0	2 (18%)	4 (36%)
Rhodes University	T2	11	0	0	0	1 (9%)	1 (9%)	0	0	2 (18%)
Rhodes University	T3	11	1 (9%)	0	0	0	3 (27%)	0	0	1 (9%)
Rhodes University	T4	11	2 (18%)	0	1 (9%)	0	4 (36%)	0	0	3 (27%)
Rhodes University	T5	11	1 (9%)	0	0	0	4 (36%)	0	0	2 (18%)
Rhodes University	T6	11	1 (9%)	0	2 (18%)	2 (18%)	8 (72%)	0	0	3 (27%)
Kenton-on-sea	T7	10	2 (20%)	0	0	0	2 (20%)	0	0	2 (20%)
Kenton-on-sea	T8	10	0	0	4 (40%)	0	2 (20%)	0	1(10%)	2 (20%)
Grahamstown west	T9	7	1 (14%)	0	0	0	1 (13%)	0	0	0
Grahamstown west	T11	7	0	0	0	0	1 (13%)	0	0	0

Note: EPEC = Enteropathogenic *E. coli*, EHEC = Enterohemorrhagic *E. coli*, EAEC = Enteroaggregative *E. coli*, EIEC = Enteroinvasive *E. coli*, NMEC = Neonatal meningitis *E. coli.*

**Table 4:** Antibiotic resistance among *E. coli* strains isolated from various rainwater tanks.

Tank ID	*n*	% Resistance										
		AP	KF	CO	GM	S	T	TS	CIP	AUG	TM	NI
T1	11	72	91	63	0	45	72	39	27	27	63	9
T2	11	72	81	36	0	27	45	45	9	54	45	18
T3	11	27	36	27	0	45	18	27	18	9	18	0
T4	11	36	90	54	0	36	36	27	0	9	27	0
T5	11	36	100	54	0	0	54	36	27	18	45	0
T6	11	45	100	45	0	18	36	36	0	9	27	0
T7	10	30	30	20	0	100	20	30	20	30	30	0
T8	10	30	100	10	0	20	10	0	0	40	0	0
T9	7	75	87	75	0	62	100	87	0	75	50	0
T11	7	0	0	0	0	0	0	0	0	40	0	0

**Table 5:** Multiple-antibiotic-resistant phenotypes of *E. coli* isolated from different rainwater tanks.

T1		T2	
MAR Phenotype	Number of Isolates	MAR Phenotype	Number of Isolates
AP-KF-CO-T	1	KF-T-NI	1
AP-KF-CO-T-TM	1	AP-KF-AUG	1
AP-KF-CO-S-T-TS-TM	1	AP-KF-NI	1
AP-KF-CO-S-T-TS-CIP-AUG-TM	1	AP-KF-S-T-TM	1
AP-KF-CO-S-T-TS-CIP-TM	1	AP-KF-CO-T-AUG	1
KF-CO-S-T-TS-AUG-TM	1	AP-KF-CO-S-T-TS-TM	1
AP-KF-CO-S-TS-CIP-TM	1	AP-KF-CO-S-T-TS-CIP-AUG-TM	1
AP-KF-CO-S-T-TS-AUG-TM	1	AP-KF-CO-T-TS-CIP-AUG-TM	1
		AP-KF-CO-S-T-TS-AUG-TM	1
**T3**		**T4**	
**MAR Phenotype**	**Number of Isolates**	**MAR Phenotype**	**Number of Isolates**
AP-KF-CO-S-TS-CIP-TM	1	KF-ST-AUG	1
AP-KF-CO-S-T-TS-TM	1	KF-T-NI	1
AP-KF-CO-S-T-TS-CIP-AUG-TM	1	AP-KF-CO-S-T-TS-TM	2
		AP-KF-CO-S-T-TS-CIP-TM	1
		AP-KF-CO-S-T-TS-CIP-AUG-TM	2
**T5**		**T6**	
**MAR Phenotype**	**Number of Isolates**	**MAR Phenotype**	**Number of Isolates**
AP-KF-CO-S-T-TS-CIP-TM	1	AP-KF-AUG	1
AP-KF-CO-T-TS-TM	1	KF-CO-S-T-TS-AUG-TM	1
AP-KF-CO-S-T-TS-CIP-AUG-TM	1	AP-KF-CO-S-TS-TM	1
KF-CO-T-TS-AUG-TM	1	AP-KF-CO-T-TS-TM-NI	1
AP-KF-CO-S-T-TS-CIP-AUG-TM	1	AP-KF-CO-S-T-TS-CIP-AUG-TM	2
**T7**		**T8**	
**MAR Phenotype**	**Number of Isolates**	**MAR Phenotype**	**Number of Isolates**
AP-KF-CO-S-T-TS-CIP-AUG-TM	1	AP-KF-T	1
AP-KF-CO-S-TS-AUG-TM	1	KF-CO-S-TS	1
AP-KF-CO-S-T-CIP-AUG-TM	1	AP-KF-CO-S-T-TS-CIP-AUG-TM	2
		AP-KF-CO-S-T-TS-AUG-TM	1
**T9**		**T11**	
**MAR Phenotype**	**Number of Isolates**	**MAR Phenotype**	**Number of Isolates**
AP-KF-CO-S-T-TS-CIP-AUG-TM	1	AP-KF-CO-S-T-TS-AUG-TM	2
AP-KF-CO-S-T-TS-AUG-TM	2	AP-KF-CO-S-T-TS-TM	1
